# A Case of Pyoderma Gangrenosum Misdiagnosed as Necrotizing Infection: A Potential Diagnostic Catastrophe

**DOI:** 10.1155/2018/8907542

**Published:** 2018-04-26

**Authors:** Medina G. Saffie, Anjali Shroff

**Affiliations:** ^1^Department of Infectious Diseases Post Graduate Training Program, McMaster University, Hamilton, ON, Canada; ^2^Department of Infectious Diseases, McMaster University, Hamilton, ON, Canada; ^3^Hamilton Health Sciences, Hamilton, ON, Canada; ^4^Department of Medicine, McMaster University, Hamilton, ON, Canada

## Abstract

In this article, we present a case of pyoderma gangrenosum (PG), misdiagnosed initially as a necrotizing infection that significantly worsened due to repeated surgical debridement and aggressive wound care therapy, almost resulting in limb amputation despite antibiotic therapy. The PG lesions improved after pancytopenia were further investigated, and the diagnosis and treatment of an underlying hematologic malignancy was initiated. The diagnosis and management of PG is challenging given the paucity of robust clinical evidence, lack of standard diagnostic criteria, and absence of clinical practice guidelines. It is imperative that clinicians recognize PG as a clinical diagnosis that must be considered in any patient with enlarging, sterile, necrotic lesions that are unresponsive to prolonged and appropriate antibiotics. Early recognition can prevent devastating sequelae such as deep tissue and bone infections associated with a chronic open wound, severe cosmetic morbidity, and potential limb amputation.

## 1. Introduction

Pyoderma gangrenosum (PG) is a rare inflammatory skin condition of unknown etiology. It can be associated with the eventual manifestation of an underlying systemic disease, most commonly hematologic malignancy or autoimmune disease [1, 2]. PG generally presents as an initial papule, pustule, or nodule after minor trauma, progressing to painful deep necrotic ulcers that wax and wane over time. It is often misdiagnosed initially as a soft tissue infection that can coincidentally improve with systemic antibiotics and wound care. Rapidly progressing PG is often erroneously diagnosed as a necrotizing infection requiring urgent surgical intervention. Further surgical debridement of lesions compounds the initial pathergic phenomenon, which accelerates the necrotic process postoperatively [3, 4]. Failure to re-evaluate the diagnosis after repeated attempts at surgical closure for nonhealing ulcers propagates the disease process and increases the risk of infectious complications with devastating patient morbidity.

## 2. Case Presentation

A 59-year-old healthy male presented for an assessment of nonhealing painful necrotic lesions of the left leg. His past medical history included anemia with intermittent pancytopenia diagnosed 10 years ago. Previous specialist hematologic assessment did not reveal an etiology. Two weeks prior, he believed that he sustained a spider bite while mowing the lawn during the month of August. The lesion began as a small papule less than 1 cm and then developed into a large blister on the left lower calf. Oral cloxacillin was initiated for one week; however, the lesion rapidly increased in size, began to ulcerate, and was extremely painful. He presented to the emergency room at a community hospital, febrile (38 degrees Celsius) with otherwise stable vitals. The documented area of necrosis measured 10 cm × 13 cm. Blood work showed ongoing pancytopenia (white blood cell count of 1.8 × 10^9^/L, hemoglobin 100 g/L, platelets 88 × 10^9^/L). He underwent urgent surgical debridement due to clinical concerns of a necrotizing process. Empiric ceftriaxone, metronidazole, and clindamycin were initiated. There was no intraoperative evidence of necrotizing fasciitis, purulence, or foul odour. Tissue cultures yielded methicillin-susceptible *Staphylococcus aureus* (MSSA), and the antibiotics were narrowed to cefazolin. Necrosis developed at the surgical margins and rapidly extended a further 3 cm over the next 3 days. A second surgical debridement was completed with the addition of a negative pressure dressing over the wound. The intraoperative tissue cultures yielded MSSA and *Pseudomonas aeruginosa*. Ciprofloxacin was added to cefazolin. During the subsequent three days, the patient developed severe calf pain with an expanding hematoma and required urgent surgical exploration. No significant abnormalities were noted. Tissue cultures yielded the same mixture of organisms. Further extension of necrosis again was observed at the surgical margin, and a fourth surgical debridement extended his wound up to the popliteal fossa and down to the ankle. Microbiology yielded commensal flora. Histopathology showed skin necrosis, ulceration and inflammation of subcutaneous tissues, and neutrophilic dermal infiltration with abscess formation. All bacterial and fungal stains were negative, and there was no evidence of vasculitis. New necrotic lesions developed on the left medial malleolus and the plantar aspect of his first metatarsophalangeal (MTP) joint. He was subsequently transferred to our centre for evaluation of an infectious etiology of progressive nonhealing necrotic skin lesions and assessment for potential limb amputation.

**Figure 1:**
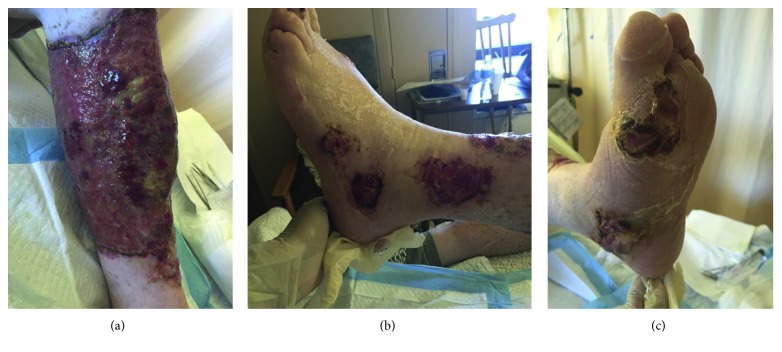
Necrotic lesions of pyoderma gangrenosum exhibiting pathergy on the left lower extremity after five surgical tissue debridement.

On initial assessment, the lesions were violaceous, painful, and necrotic with raised, irregular borders. The largest lesion extended circumferentially from the left tibial tuberosity to the ankle. Satellite lesions were noted at the medial and lateral malleoli and the base of the first MTP joint (Figure 1). Investigations showed ongoing pancytopenia (leukocytes 2.3 × 10^9^/L, hemoglobin 72 g/L, and platelets 64 × 10^9^/L). His C-reactive protein level was 225 mg/L. A nuclear bone scan indicated osteomyelitis of the left first MTP joint. A bone marrow biopsy showed pancytopenia with possible hairy cells. Flow cytometry revealed a pattern consistent with hairy cell leukemia (HCL).

**Figure 2:**
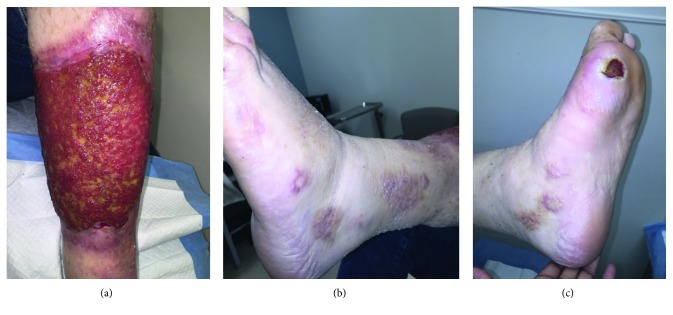
Pyoderma gangrenosum lesions 6 months after treatment of underlying hematologic malignancy and use of antibiotics for osteomyelitis of the first MTP.

Based on the clinical course of leg lesions exhibiting a pathergic phenomenon in response to skin trauma and nonresponse to multiple adequate courses of antimicrobial therapy and pathology showing sterile neutrophilic infiltration, combined with diagnosis of underlying hematological malignancy, we diagnosed him clinically with pyoderma gangrenosum. All further debridement was suspended, and negative pressure dressings were stopped. Due to the open nature of the wound, imaging consistent with osteomyelitis and impending chemotherapy, empiric piperacillin-tazobactam and vancomycin were initiated. A bone biopsy of the left MTP grew *E.* coli (Amp C) and *P. aeruginosa.* Antibiotics were switched to ertapenem and ciprofloxacin. He received seven days of cladribine chemotherapy plus prednisone for HCL and continued antibiotics for osteomyelitis of his left MTP. Six months after diagnosis of PG, the lesions showed significant clinical improvement, but severe scarring (Figure 2).

## 3. Discussion

Historically, the etiology of PG was erroneously believed to be infectious [5]. Today, PG is classified as a neutrophilic dermatosis, as histological examination exhibits predominantly neutrophilic infiltrates, without evidence of infection [2, 6, 7]. Although the underlying pathogenesis remains unclear, increasing evidence points to autoimmune mechanisms of dysregulated inflammation [7, 8]. Neutrophilic dysfunction, systemic inflammation, and associated genetic factors are all involved in the formation of PG ulcers [7]. Previously, no criteria consistently or reliably distinguished PG from necrotizing soft tissue infections, particularly in the absence of systemic diseases associated with PG (Table 1) [9, 10]. Recently, a validated set of criteria have been published by Maverakis et al., where one major criterion (skin biopsy demonstrating neutrophilic infiltration) and 4 of 8 minor criteria could be used to diagnose PG with high sensitivity and specificity (86% and 90% resp.) [11]. Although current clinical practice supports PG as a diagnosis of exclusion, if there is a high degree of suspicion in the appropriate clinical situation, these criteria could serve as more robust guidance for clinicians [10–12].

Pathergy is an important feature that, if present, can support the diagnosis of PG. It is defined as the development of skin lesions that resist healing after tissue injury [14, 15]. A history of minor skin trauma is usually the inciting event, as exhibited in our case presentation. The lesion classically begins as a pustule, vesicle, or nodule that progresses (from days to weeks) into a painful ulcer or erosion with raised borders [15]. Lesions can spontaneously heal without intervention or coincidentally with empiric antibiotic treatment. They can remain quiescent for months to years and reemerge with trivial trauma or for no apparent cause [15]. Often patients believe they have been bitten by a venomous spider or insect and seek early medical attention. Early aggressive tissue debridement generates more trauma and can compound the initial pathergic response [3, 4], as was the case in our patient.

The clinical course of PG is not predictable. Healing is variable and highlights the requirement for an individualized approach to diagnosis and treatment. First, skin biopsies for histology and microbiology are critical to narrow the differential diagnosis [16]. Infections that can mimic PG include atypical mycobacterial ulcers, cutaneous tuberculosis, cutaneous leishmaniasis, sporotrichosis, and other deep fungal infections shown in Table 2 [6, 14, 17]. The noninfectious differential is broad and should also be considered. It includes vasculitis, thrombophilias, cutaneous malignancies and drug-induced conditions also shown in Table 2 [6, 14, 17]. An appropriate exposure history can help narrow the possible infectious etiologies but does not negate the need for skin biopsy. Histopathology of PG usually shows nonspecific inflammation with intradermal abscess formation [6, 9, 12, 16]. Initial wound cultures yielding skin flora such as *S. aureus* are often erroneously considered the culprit, which occurred in our patient's example. In true PG, targeted antimicrobial therapy eventually fails, and lesions can progressively enlarge with further debridement. Subsequent tissue cultures on antibiotics begin to select for gram-negative bacteria, including *P. aeruginosa*. Antibiotic exposure and persistent deep, open wounds predispose to a superimposed infection with more resistant organisms, as was demonstrated in our patient.

Treatment of PG remains challenging as no single effective therapeutic regimen or consensus guideline exists. Initial investigation for associated underlying systemic disease is of crucial importance as treating this can hasten resolution of PG as presented in our case [10]. For mild PG disease such as single or superficial lesions, conventional evidence-based first-line treatments involve topical medications such as high-potency corticosteroids or calcineurin inhibitors [7]. The efficacy of these were assessed in a prospective cohort study which found that 44% of lesions healed at 6 months and 15% of those had recurrent lesions [18]. For severe PG lesions, first-line systemic therapy includes systemic corticosteroids or cyclosporine [19]. Evidence from a randomized control trial demonstrated that at 6 months both therapies were equivalent with respect to healing response (47% for oral prednisolone and 47% for cyclosporine) [20]. About one-third of patients had recurrences in both groups [20]. Therefore, first-line systemic therapy for severe PG should be chosen based on patient tolerability and side effect profiles. There is a paucity of data to guide clinical decision-making when considering second-line systemic therapies (methotrexate, azathioprine, mycophenolate mofetil, cyclophosphamide, dapsone, thalidomide, and intravenous immunoglobulins) and are mainly used as steroid-sparing agents for maintenance therapy or in combination with first-line agents for refractory disease as reviewed by Patel et al. [19]. However, these drugs are not without significant side effects and toxicities as reviewed by Feldman et al. [21].

Improved understanding of the molecular mechanisms underlying dysregulated inflammation in PG has expanded treatment options to include biologics. For example, Marzano et al. demonstrated in their study using skin biopsies that the inflammation associated with PG involves increased expression of cytokines and chemokines, particularly tumor necrosis factor (TNF)-*α*, interkeukin (IL)-1*β*, and IL-17 [22]. This proof-of-concept study supports using therapies that target dysregulated autoinflammation to treat PG. Currently, TNF-*α* inhibitors such as infliximab are established therapies used to successfully treat inflammatory bowel disease and can improve PG associated with the systemic disease [23]. IL-1 is a key inflammatory mediator associated with syndromic and nonsyndromic presentations of PG that can trigger release of chemokines that are involved in neutrophil recruitment and activation [8]. Anakinra and canakinumab are both therapies that block IL-1 and have been used to treat refractory PG with the rationale of blocking the autoinflammatory cascade as discussed in detail by Garcovich et al. [13, 24]. Despite the promising advances in biologic targeted therapies, the vast majority of treatment data available today are based on case series and case reports that vary in treatment combinations, dosing, duration, and outcomes. This makes it impossible to compare efficacy of regimens. Further clinical trials with these and other biologic agents that target the inflammatory pathway of PG will enhance treatment regimens for severe and refractory disease.

A personalized, holistic approach to treatment cannot be overstated, particularly with respect to optimal wound care, infection prevention, and further surgical intervention [25, 26]. Assessment by a wound care specialist is critical for healing. Multidisciplinary discussions are required when considering any debridement of necrotic tissue, skin grafting, or negative pressure dressings due to increased risks of pathergy and infection.

## 4. Conclusion

In summary, we present a case of PG misdiagnosed as a necrotizing infection with multiple surgical interventions resulting in a pathergic phenomenon. Successful treatment of the underlying hematologic malignancy was associated with improvement, but the delay in recognition of PG resulted in significant infectious complications, severe cosmetic morbidity, and potential limb amputation. PG must be considered in any patient with enlarging, sterile, necrotic lesions that are unresponsive to prolonged antibiotics targeted at skin flora. Inflammation is the basis of the disease process, but pathophysiological mechanisms and subsequent targeted therapies for PG with more robust studies are yet to be elucidated. Treatment should focus on both diagnosis and management of an underlying systemic disease with a multidisciplinary approach to wound care and prevention of secondary infection. The recent development of validated diagnostic criteria and availability of biologic agents will optimize diagnosis and treatment options for severe and refractory PG.

## Figures and Tables

**Table 1:** Underlying systemic diseases associated with pyoderma gangrenosum.

Inflammatory bowel disease—Crohn's disease, ulcerative colitis
Hematologic disorders
Hematologic malignancy
Multiple myeloma
Myelodysplasia
Polycythemia vera
Arthritis
Rheumatoid arthritis
Seronegative arthritis
Inherited autoinflammatory syndromes [13]
PAPA
PAPASH
SAPHO

AML, acute myeloid leukemia; PAPA, pyogenic arthritis, pyoderma gangrenosum, and acne; PAPASH, pyogenic arthritis, pyoderma gangrenosum, acne, and hidradenitis suppurativa; SAPHO, synovitis, acne, pustulosis, hyperostosis, and osteitis.

**Table 2:** Etiologies of skin lesions that can mimic pyoderma gangrenosum.

Infectious	Noninfectious
Atypical mycobacteria	Vascular
*Mycobacterium marinum*	Polyarteritis nodosa
*Mycobacterium ulcerans*	ANCA-associated vasculitis
Tuberculosis (cutaneous)	Cryoglobulinemic vasculitis
Leishmaniasis (cutaneous)	Venous stasis
Ecthyma gangrenosum	Thrombophilia
Anthrax (cutaneous)	Antiphospholipid syndrome
Syphilitic gumma	Malignancy
Deep fungal infections	Squamous/basal cell carcinoma
Sporotrichosis	Cutaneous T-cell lymphoma
Zygomycosis	Drug-induced/toxin
Aspergillosis (primary cutaneous)	Cutaneous lupus (hydralazine, TNF-alpha inhibitors)
Penicilliosis (HIV with CD4 < 100/*μ*L)	Hydroxyurea
Injection drug use with secondary infection	Venomous bite (brown recluse spider)
